# Chaotic Multiquenching Annealing Applied to the Protein Folding Problem

**DOI:** 10.1155/2014/364352

**Published:** 2014-03-20

**Authors:** Juan Frausto-Solis, Ernesto Liñan-García, Mishael Sánchez-Pérez, Juan Paulo Sánchez-Hernández

**Affiliations:** ^1^Universidad Politécnica del Estado de Morelos Boulevard, Cuauhnáhuac 566, 62660 Jiutepec, Mexico; ^2^Universidad Autónoma de Coahuila Boulevard, Venustiano Carranza s/n, 25280 Saltillo, Mexico; ^3^Computational Genomics Research Program, Center for Genomic Sciences, Universidad Nacional Autónoma de México, Avenida Universidad s/n, 62210 Cuernavaca, Mexico; ^4^Instituto Tecnológico y de Estudios Superiores de Monterrey, Autopista del Sol, 62790 Xochitepec, Mexico

## Abstract

The Chaotic Multiquenching Annealing algorithm (CMQA) is proposed. CMQA is a new algorithm, which is applied to protein folding problem (PFP). This algorithm is divided into three phases: (i) multiquenching phase (MQP), (ii) annealing phase (AP), and (iii) dynamical equilibrium phase (DEP). MQP enforces several stages of quick quenching processes that include chaotic functions. The chaotic functions can increase the exploration potential of solutions space of PFP. AP phase implements a simulated annealing algorithm (SA) with an exponential cooling function. MQP and AP are delimited by different ranges of temperatures; MQP is applied for a range of temperatures which goes from extremely high values to very high values; AP searches for solutions in a range of temperatures from high values to extremely low values. DEP phase finds the equilibrium in a dynamic way by applying least squares method. CMQA is tested with several instances of PFP.

## 1. Introduction

DNA is a molecule that contains genetic instructions, which are used in protein synthesis process [1]. This molecule has a complete set of hereditary information of any organism. DNA is formed by four different nucleotides, Adenine identified by the letter *A*, Cytosine identified by the letter *C*, Guanine identified by the letter *G*, and Thymine identified by the letter *T*. This molecule is divided into genes; each gene is a sequence of nucleotides that can express a functional protein. The transcription process of DNA creates an RNA molecule, which generates proteins. A protein is a linear polypeptide of amino acids, which are joined by peptide bonds. The atoms of a protein are arranged in a three-dimensional structure geometric model. In principle, function and structure of a protein are determined by its amino acids sequence. A functional protein is conformed in a geometrical model with a global minimum energy [2]; however, there are some exceptions [3]. This structure is usually named native structure (NS). The free energy of a conformation depends on the interaction among the atoms and their relative positions; normally, this energy can be calculated using torsion angles and the distance among atoms.

A protein can take consequently many different conformational structures from its primary structure to its native structure [4]. Therefore, computational methods are currently designed in order to find the optimal solution, which has the minimal free energy and determines the NS. The computational problem involved to find the NS is known as protein folding problem (PFP). Because PFP is a NP problem [5], metaheuristic methods avoid the generation of all possible states of the protein [6]. A particular class of these methods is known as *Ab*-*Initio*. In other words, *Ab*-*Initio* methods search for NS only using protein sequence amino acids.

New heuristic methods are used to solve PFP, where simulated annealing (SA) [7, 8] is one of the most successful. However, in order to generate high-quality solutions for PFP, new and more efficient SA should be designed [9]; one of them is named Multiquenching Annealing algorithm (MQA) [10]. This algorithm uses two phases. The first one or quenching phase applies a fast cooling rate to reach a fast solution. In contrast, the second phase applies a slow cooling rate in order to obtain a high-quality solution.

In this paper, a new approach named Chaotic MultiQuenching Annealing (CMQA) for PFP is presented. CMQA has three phases. The first one applies a quenching process and chaotic functions in several subphases. The second phase implements an annealing process. In the third phase, the stochastic equilibrium is detected by using least squares method.

## 2. Materials and Methods

In this section the protein folding problem is briefly described and the next methods are explained: SA, MQA, and CMQA. Then chaotic local search (CLS) is introduced and compared with those algorithms trough a set of small proteins.

### 2.1. Protein Folding Problem

Native structure prediction of a protein is an enormous challenge in the computational biology domain [11, 12]. PFP is an interdisciplinary problem which involves molecular biology, biophysics, computational biology, and computer science [13]. In the case of *Ab*-*Initio*, NS prediction requires different mechanisms that lead the searching process to a unique biological three-dimensional structure. This process only requires amino acids' sequence. There is an extremely large space of possible conformations of the protein; the size of this space depends on the length of the sequence of amino acids [4].

The function of a protein directly is related to its three-dimensional structure, and misfolded proteins can cause a variety of diseases [14–19]. In addition, PFP is analyzed in protein engineering area [20] where proteins are designed and constructed with desired functions and structures. PFP can be solved by different combinatorial optimization algorithms [21]. An objective function of PFP would be optimized by finding the native structure of a protein. PFP requires the following information:

- a sequence of *n* amino acids *a*_1_, *a*_2_, …, *a*_*n*_ that represents the primary structure of a protein;
- an energy function, *f*(*σ*_1_, *σ*_2_,…, *σ*_*m*_), which represents the free energy. The variables *σ*_1_, *σ*_2_,…, *σ*_*m*_ represent the *m* dihedral angles.

The solution of this problem is to find the native structure such that f*(*σ*_1_, *σ*_2_,…, *σ*_*m*_) represents the minimal energy value. The optimal solution σ* = (*σ*_1_, *σ*_2_,…, *σ*_*m*_) defines the best three-dimensional configuration. Force fields are used to represent the energy of a protein [22]; some of the most common are AMBER [23], CHARMM [24], ECEPP/2, and ECEPP/3 [25]. These fields compute some energy components, for example, the electrostatic energy [25], the torsion energy [23], the hydrogen bond energy, and the Lennard-Jones energy [26].

Simulated annealing algorithm has generated very good results for PFP [9, 27–29]. This method has been used in many combinatorial optimization problems [9, 10, 30–32]. However, SA has a low convergence feature and requires too much execution time. Thus, it is convenient to develop new SA strategies for improving its effectiveness.

### 2.2. Chaotic Multiquenching Annealing Algorithm

#### 2.2.1. General Description

**Figure 1:**
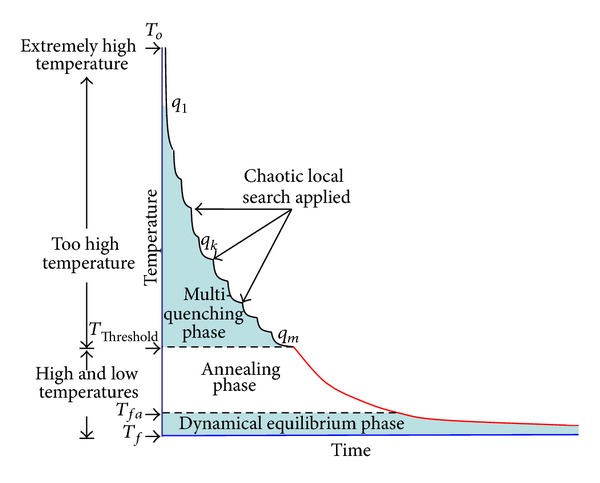
CMQA phases.

The Chaotic Multiquenching Annealing (CMQA) introduced in this paper is composed of three phases as it is shown in Figure 1: (i) multiquenching phase (MQP) applies several quenching processes, all of which implement a chaotic local search at the end of each stage; (ii) annealing phase (AP) is a classical simulated annealing process; and (iii) dynamical equilibrium phase (DEP) detects the stochastic equilibrium in a dynamical way using a regression method. MQP is applied from extremely high temperature to very high values. This phase applies a very fast cooling function to decrease the temperature parameter. MQP is executed from *T*_0_ until *T*_threshold_. After this phase, AP is executed until a final threshold temperature (*T*_*fa*_), which is close to the final temperature of the whole algorithm. AP develops an exploration of the solution space with a very slow temperature's decrement. Finally, DEP detects the final temperature *T*_*f*_ by using an efficient implementation of the least squares method.

All CMQA's phases apply a cooling function (1), which is similar to that applied in the classical simulated annealing algorithm. The initial and final temperatures (*T*_0_ and *T*_*f*_) can be determined experimentally and/or analytically. The *α* parameter is a decrement temperature factor; it is less than one and greater than a certain value (close to 0.7) as follows:

(1) $$\begin{array}{c} T_{k+1}=\alpha T_{k},\;k=0,1,2,\ldots ,\;\;0.7\leq \alpha <1. \end{array}$$

#### 2.2.2. Multiquenching Phase

**Figure 2:**
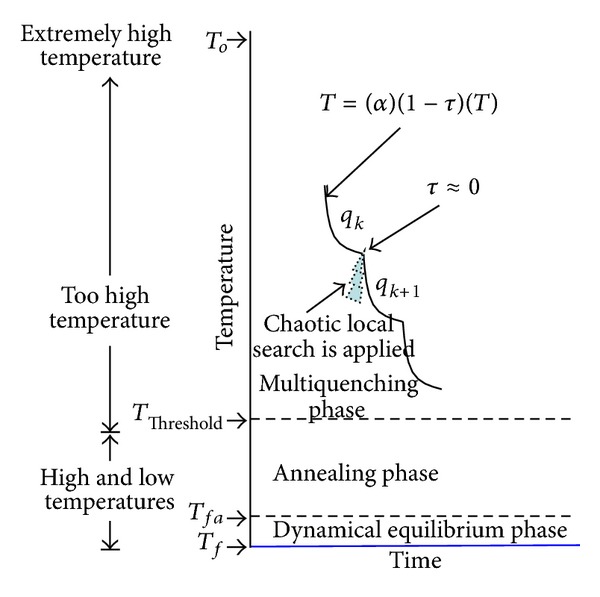
Chaotic local search.

MQP has several subphases (see Figure 2). It starts at extremely high initial temperature (*T*_0_) and it is finished when a threshold temperature (*T*_Threshold_) is reached. MQP uses the cooling function given by (2) and (3). In this case, the temperature is decreased by using *α*_Quenching_ and *τ* parameters. *α*_Quenching_ parameter is in the range (0,1), and it defines how fast each MQP's subphase is decreased. A very low value *α*_Quenching_ will decrease the temperature very fast. The *τ* parameter is ranged in (0,1), and it defines a quadratic decrement of the temperature. Notice that *τ* converges to zero, and then (2) is equivalent to (1) as follows:

(2) $$\begin{array}{c} T_{k+1}=\alpha_{\text{Quenching}}\left(1-\tau \right)T_{k},\;k=0,1,2,\ldots ,\;\;0.7\leq \alpha <1, \end{array}$$

(3) $$\begin{array}{c} \tau =\tau^{2},\;0<\tau <1. \end{array}$$

The transition between two subphases is based on *τ* parameter. It occurs when *τ* converges to zero (*τ* ≈ 0). In this transition, a chaotic local search (CLS) is started. When CLS is finished, the new MQP subphase (i.e., another quenching process) is started, and *τ* is set to its initial value. This process continues until the temperature *T*_Threshold_ is reached. Actually, this temperature corresponds to the initial temperature of a classical SA algorithm. Therefore, MQP is an additional search procedure that looks for improving the quality solution, even though the execution time is increased. An alternative approach is to increment the iterations' number of the classical SA. However, in this alternative, the quality solution is not significantly improved according to previous experimentation.

Algorithm 1 shows the MQP's pseudocode. In the setting section, MQP's parameters are established. The initial temperature *T*_0_ is defined according to a tuning method [33], while *α*_Quenching_ and *τ* parameters experimentally are set (in this case 0.85 and 0.90, resp.). MQP generates a random initial solution *S*_*i*_ (with an energy *E*(*S*_*i*_)) at the temperature *T*_0_, which determines an initial minimal solution candidate (*S*_min⁡_). Two main cycles can be observed in this algorithm. The first one (external cycle) controls the temperature, which is decreased by applying geometric function (2). The other cycle (or metropolis cycle) generates new solutions *S*_*j*_ by using a perturbation function. This function is a classical probabilistic distribution at the beginning of the process, which is different to that used at the end of the algorithm when a chaotic search procedure is used. In the internal cycle, *S*_*j*_ is always accepted if it is better than a previous solution. When a new solution does not improve the previous one, it is accepted or rejected with the Boltzmann distribution. When *S*_*j*_ is accepted, it replaces the previous solution *S*_*i*_ (i.e., *S*_*i*_ = *S*_*j*_). When a new accepted solution *S*_*i*_ is better than the current minimal solution *S*_min⁡_, it is replaced by *S*_*i*_ (i.e., *S*_min⁡_ = *S*_*i*_). Each time a metropolis cycle is finished, the *τ* parameter is updated according to (3), and when its value converges to zero, a chaotic local search (CLS) is executed. Once CLS (explained in Section 2.2.4) is finished, the *τ* parameter retakes its initial value and a new MQP subphase is started. In this case, a new temperature is calculated using (2), and the process continues until the *T*_Threshold_ temperature is obtained.

The parameter *T*_0_ is set to an initial value and is assigned to *T* (see line 4). The threshold temperature (*T*_Threshold_) is set to an initial value (see line 5). *α*_Quenching_ and *γ* are set to initial value (see line 6). *S*_*i*_ is set to initial solution. *E*(*S*_*i*_) is calculated, which represents the energy of *S*_*i*_ (see line 8). *S*_min⁡_ is set to *S*_*i*_. The energy of *S*_min⁡_ is set to *E*(*S*_*i*_). The external cycle is started (see line 11), and this is finished at line 30. The metropolis cycle is started within the temperature cycle (see line 12), and this cycle is finished at line 23. Within this cycle, *S*_*j*_ is created by applying a uniform perturbation (see line 13). The difference of energies between *E*(*S*_*j*_) and *E*(*S*_*i*_) is calculated (see line 14). If this difference is less than zero (see line 15), then the *S*_*j*_ is accepted (see line 16). Then, this solution *S*_*j*_ is assigned to *S*_*i*_. If this difference is greater than zero, then the Boltzmann probability is calculated by using *e* ^−(difference/*T*)^ (see line 17). If this probability is greater than a random value between 0 and 1 (see line 17), then the *S*_*j*_ is accepted (see line 18). Then, this solution *S*_*j*_ is assigned to *S*_*i*_. If *S*_*i*_ is less than *S*_min⁡_ (see line 20), then *S*_*i*_ is assigned to *S*_min⁡_ (see line 21). After the metropolis cycle is finished, the variable *γ* is updated by (3) (see line 24). If *γ* is very close to zero (see line 25), then *γ* is set to initial value (see line 26), and the chaotic search is called (see line 27). The temperature value *T* is set by applying (2) (see line 29).

#### 2.2.3. Setting the Temperature Range

CMQA uses an analytical tuning method to determine the initial and final temperature [33]. This method is based on the acceptance probability of the solutions. At the beginning, the probability of accepting a new solution is very close to one. This occurs at extremely high temperatures; consequently, the deterioration of the cost function is maximal. Therefore, the initial temperature *T*_0_ is associated with the maximal deterioration Δ*Z*_max⁡_. On the other hand, the probability of a new solution is very close to zero at very low temperatures; in this case, the deterioration of cost function is minimal. Thus, the final temperature *T*_*f*_ is associated with the minimal deterioration Δ*Z*_min⁡_. The acceptance probability based on Boltzmann distribution is defined by (4). At extremely high temperatures, this equation leads to (5). On the other hand, at the end of the process, the final temperature is obtained by (6) as follows:

(4) $$\begin{array}{c} P\left(\Delta Z\right)=\exp\left(\frac{-\Delta Z}{T}\right), \end{array}$$

(5) $$\begin{array}{c} T_{0}=\frac{-\Delta Z_{\max}}{\ln\left(P\left(\Delta Z_{\max}\right)\right)}, \end{array}$$

(6) $$\begin{array}{c} T_{f}=\frac{-\Delta Z_{\min}}{\ln\left(P\left(\Delta Z_{\min}\right)\right)}. \end{array}$$

Actually, CMQA uses the final temperature only as a guide to detect stochastic equilibrium at dynamical equilibrium phase. This phase is a special process based on least squares method during the last phase of CMQA. DEP is started some cycle before *T*_*f*_ and is explained in Section 2.2.6.

#### 2.2.4. Chaotic Local Search

In order to avoid falling into local optima, CMQA applies CLS procedure at very high temperatures. As it is shown in Algorithm 2, this process has only a search cycle; *S*_min⁡_ solution is improved by a chaotic function *f*(*x*). This function is named chaotic perturbation in the pseudocode of Algorithm 2. The purpose of this chaotic function is to improve the possibility of escape from any local optimum. In CLS, *S*_*j*_ solution is generated by applying a chaotic perturbation to *S*_min⁡_; when *S*_*j*_ is better than *S*_min⁡_, then *S*_min⁡_ is replaced by *S*_*j*_. Thus, *S*_min⁡_ solution is improved after several iterations (*M*_chaot_). Generally, CLS improves *S*_min⁡_ when *M*_chaot_ is equal to the number of instance's variables. The current solution *S*_*i*_ is assigned to *S*_aux_ (see line 3). The minimal solution *S*_min⁡_ is assigned to *S*_*i*_ (see line 4). The FOR statement is started at line 5, and it is finished at line 11. Within this FOR statement, the solution *S*_*j*_ is created by applying a chaotic perturbation to *S*_*i*_ (see line 6). If solution *S*_*j*_ is better than *S*_min⁡_, then *S*_*j*_ replaces *S*_min⁡_ (see line 8). The solution *S*_min⁡_ is assigned to *S*_*i*_ (see line 10). After FOR statement is finished, *S*_aux_ is assigned to *S*_*i*_.

#### 2.2.5. Annealing Phase

The annealing phase (AP) corresponds to the classical simulated annealing algorithm and it is shown in Algorithm 3. When CMQA reaches its threshold level (*T*_Threshold_), AP phase is started with the cooling function (1) using *α*_Quenching_ as decrement temperature factor. As it is known, AP phase contains two cycles, as it is common in classical SA. This pseudocode uses the same notation previously explained in Section 2.2.2.

#### 2.2.6. Dynamical Equilibrium Based on Least Squares Method

CMQA algorithm dynamically finds the equilibrium by using least squares method. In order to obtain better solutions for PFP, this approach is applied after AP phase. Let (*x*, *E*_*i*_) be a set of *n* points with *i* = 1,2,…, *n*.   *E*_*i*_ represents the energy of protein at *x*_*i*_ point. The goal is to find a straight line, which is defined as *f*(*x*_*i*_) = *ax*_*i*_ + *b* that approximates the set of points, where *a* represents the slope of the straight line, and it is calculated by applying least squares method. The *b* parameter represents the intersection with axis. These parameters are calculated by

(7) $$\begin{array}{c} a=\frac{n\sum_{i=1}^{n}x_{i}E_{i}-\left(\sum_{i=1}^{n}x_{i}\right)\left(\sum_{i=1}^{n}E_{i}\right)}{n\sum_{i=1}^{n}x_{i}^{2}-{\left(\sum_{i=1}^{n}x_{i}\right)}^{2}},\\ b=\frac{\left(\sum_{i=1}^{n}E_{i}\right)\left(\sum_{i=1}^{n}x_{i}^{2}\right)-\left(\sum_{i=1}^{n}x_{i}\right)\left(\sum_{i=1}^{n}\left(x_{i}E_{i}\right)\right)}{n\sum_{i=1}^{n}x_{i}^{2}-{\left(\sum_{i=1}^{n}x_{i}\right)}^{2}}. \end{array}$$

It is easy to show that the slope of the straight line can be calculated by

(8) $$\begin{array}{c} a=\frac{12\sum_{i=1}^{n}x_{i}E_{i}-6\left(n-1\right)\sum_{i=1}^{n}E_{i}}{n^{3}-n}. \end{array}$$

CMQA determinates the dynamical equilibrium. This condition is obtained when the slope (*a*) of the straight line is very close to zero.

## 3. Results and Discussion

CMQA is tested with five instances of PFP (see Table 1). These instances have different sequence's lengths and different number of variables (dihedral angles). The smallest sequence is Met^5^-enkaphalin, which has five amino acids and 19 variables. The largest sequence is a hypothetical protein (CASP T0281), which has 90 amino acids and 458 variables. The proinsulin instance has 31 amino acids and 132 variables; the 2K5E (CASP T0549) has 73 amino acids and 343 variables. The instance* Bacillus subtilis* (CASP T0335) has 85 amino acids and 450 variables. The dihedral angles used in the simulations were phi (Φ), psi (Ψ), omega (*ω*), and Chi (*χ*).

**Figure 3:**
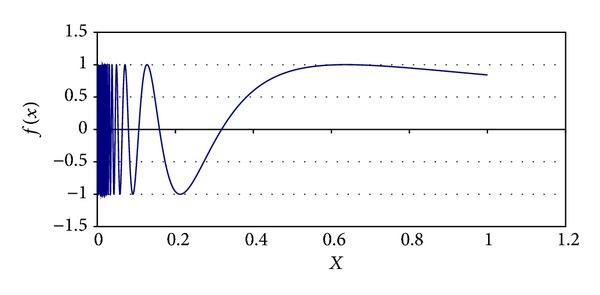
Chaotic function (9).

**Figure 4:**
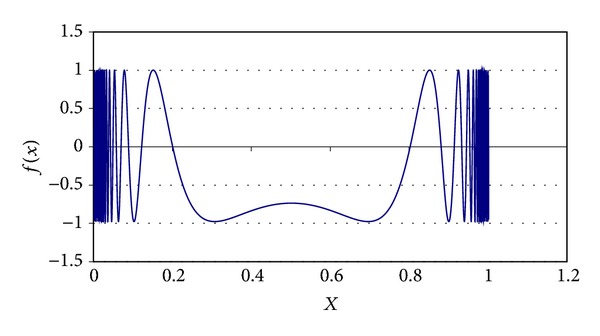
Chaotic function (10).

**Figure 5:**
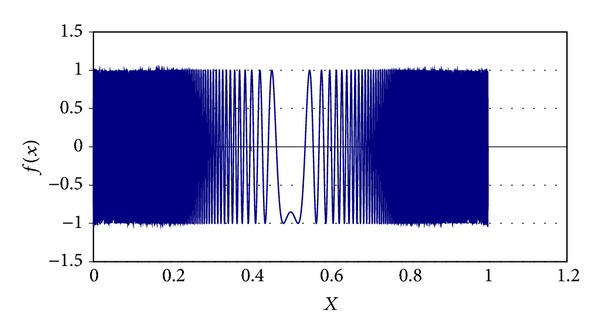
Chaotic function (11).

**Figure 6:**
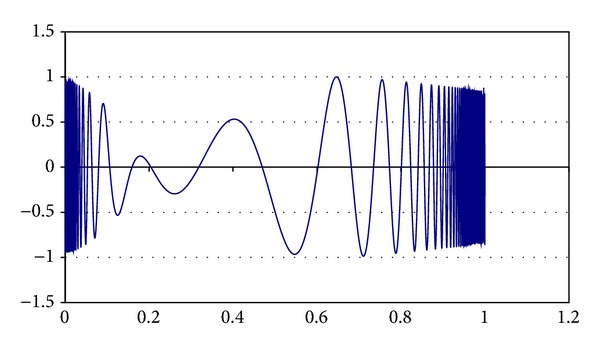
Chaotic function (12).

Some parameters of MQP phase were determined experimentally. For example, the *α*_Quenching_ is set to 0.85 value; the initial value for *τ* is 0.90, and its final value is 0.0009. Different chaotic functions were tested for generating PFP solutions. These chaotic functions are (9), (10), (11), and (12). These equations are graphically shown in Figures 3, 4, 5, and 6, respectively. In AP phase, *α*_Annealing_ was fixed from different values taken from the range [0.75, 0.95] as follows:

(9) $$\begin{array}{c} f\left(x\right)=\sin\left(\frac{1}{x}\right), \end{array}$$

(10) $$\begin{array}{c} f\left(x\right)=\sin\left(\left(\frac{1}{x}\right)\left(\frac{1}{1-x}\right)\right), \end{array}$$

(11) $$\begin{array}{c} f\left(x\right)=\sin\left(\left(\frac{100}{x}\right)\left(\frac{1}{1-x}\right)\right), \end{array}$$

(12) $$\begin{array}{c} f\left(x\right)=\sin\left(\frac{1}{x}\right)*\sin\left(\frac{5}{1-x}\right). \end{array}$$

**Figure 7:**
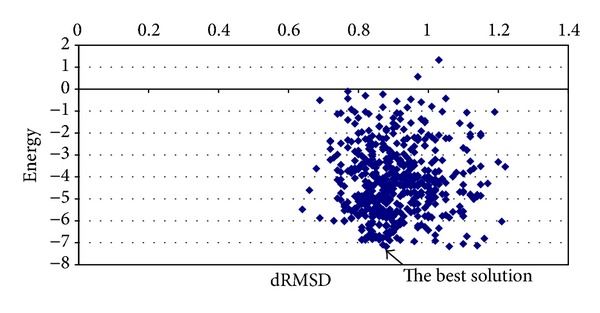
Graphic of average energy and dRMSD (Met^5^-enkaphalin instance).

The results obtained are shown in Tables 2 to 6, which include information about the average energy of each protein (kcal/mol), its average processing time (minutes), and dRMSD. These results are grouped by *α*_Annealing_ values for each chaotic function. For Met^5^-enkaphalin, the results are shown in Table 2. The best average solution for this protein was obtained by applying *α*_Annealing_ = 0.95 and the chaotic function number 12; the best average energy was −5.4390 kcal/mol, with a processing time equal to 1.1191 minutes and dRMSD equal to 0.8913.  Figure 7 shows the best solution with a dRMSD close to 0.88 and energy value equal to −7.1804 kcal/mol.

**Figure 8:**
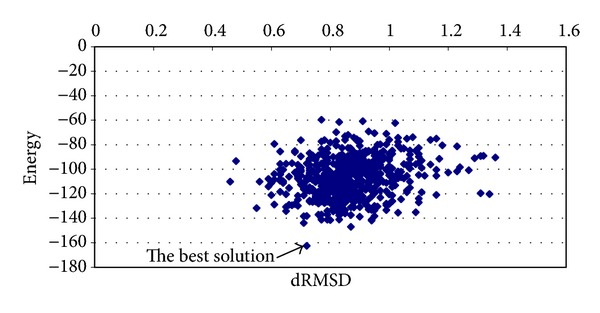
Graphic of average energy and dRMSD (proinsulin instance).

**Figure 9:**
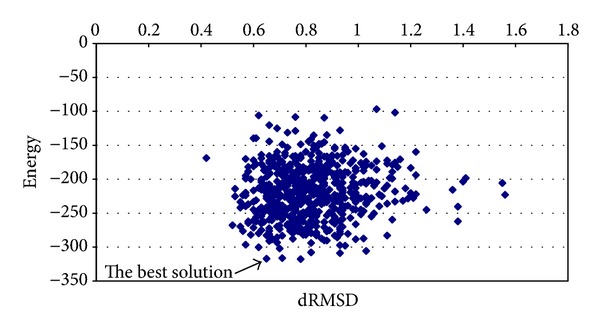
Graphic of average energy and dRMSD (T0549 instance).

The results obtained for proinsulin are shown in Table 3. The best average solution for this protein was obtained by applying chaotic function number 12. The best solution has −126.9481 kcal/mol obtained with a processing time equal to 38.0507 minutes and dRMSD equal to 0.8233. Notice that the best results are obtained with high values. In Figure 8, the best solution is shown which has −162.5686 kcal/mol and a dRMSD equal to 0.72. The results obtained for T0549 instance are shown in Table 4. The solution with the best average energy is −269.6413 kcal/mol with processing time equal to 288.8558 minutes and dRMSD value of 0.72. Again, the best solution corresponds to the highest value of *α*_Annealing_ equal to 0.95. In this case, chaotic function number 10 provided the best results. The graphic of average energy versus dRMSD is shown in Figure 9. The solution with the best quality solution has an energy value of −317.1750 kcal/mol with dRMSD value of 0.65.

**Figure 10:**
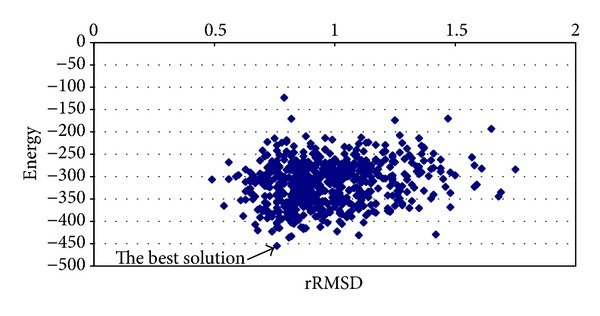
Graphic of average energy and dRMSD (T0335 instance).

The results obtained for T0335 instance are shown in Table 5. The best average solution for this instance is obtained by applying chaotic function number 11. The best average energy is −377.6919 kcal/mol with a processing time equal to 379.8146 minutes and RMSD value of 0.9787. In Figure 10, the graphic of average energy and dRMSD is shown. There is a solution with high quality (see arrow on graphics). The energy value is −455.0870 kcal/mol with dRMSD value of 0.76.

**Figure 11:**
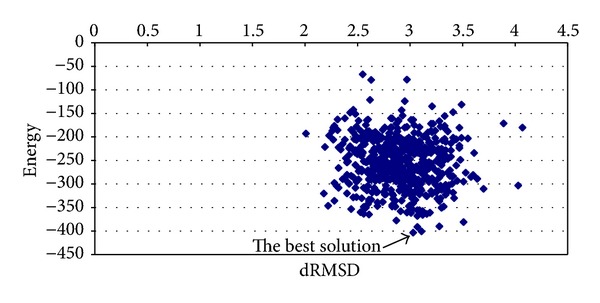
Graphic of average energy and dRMSD (T0281 instance).

The results obtained for T0281 are shown in Table 6. The best average solution for this protein is obtained by applying chaotic function number 13. The best average energy is −319.9603 kcal/mol with a processing time equal to 554.1053 minutes and dRMSD value of 2.9197. In Figure 11, the graphic of average energy and dRMSD is shown. In these graphics, all energy of T0281 calculated by CMQA is plotted. There is a solution with high quality (see arrow on graphics). The energy value is −403.3333 kcal/mol with dRMSD value of 3.03.

In order to compare the CMQA with other implementations, two algorithms were designed. The Multiquenching Annealing with dynamical equilibrium phase (MQA plus DEP) and classical simulated annealing were implemented. The results obtained are shown in Table 7. In general, CMQA obtained high-quality solutions in comparison with other implementations.

## 4. Conclusions

In this paper, a new algorithm for protein folding problem named Chaotic Multiquenching Annealing or CMQA is proposed. In order to escape from local optima, this algorithm applies a chaotic function in each subphase of quenching. In addition, a very fast cooling function is applied in order to decrease the temperature values and change the subphase. During the multiquenching phase, solutions of PFP are generated in order to explore the solution space in a very fast way. An annealing phase is applied after the multiquenching phase. In this phase, a very slow cooling function is used in order to decrease temperature values. Besides, the annealing phase searches for solutions from high to lower temperatures. The last phase of CMQA is named dynamical equilibrium phase, in which slope values of energy are calculated using least squares method. The CMQA disadvantage is related to the processing time, which is increased in order to obtain high-quality solving. Therefore, processing time is sacrificed to achieve quality in solving the protein folding problem.

## Figures and Tables

**Algorithm 1:**
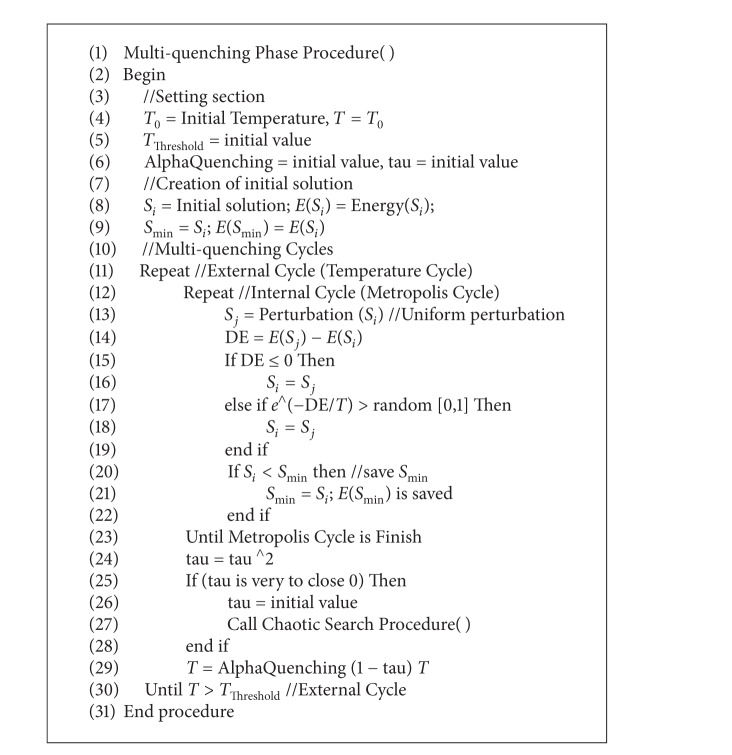
MQP pseudocode.

**Algorithm 2:**
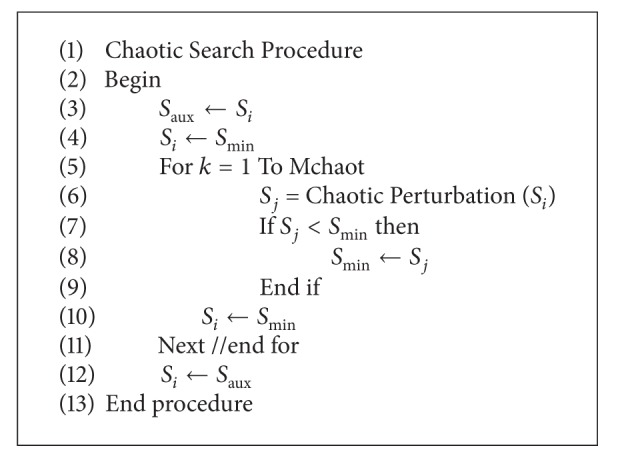
CLS pseudocode.

**Algorithm 3:**
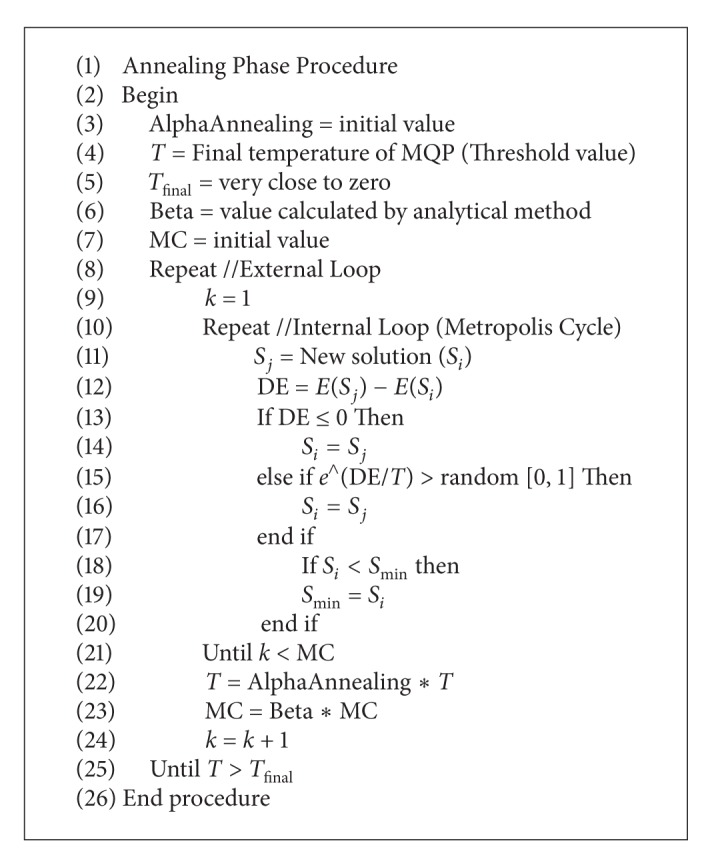
AP pseudocode.

**Table 1:** Instances of PFP.

Instance of PFP	Amino acids	Mchaot (number of variables)
Met^5^-enkaphalin	5	19
Proinsulin	31	132
T0549	73	343
T0335 (*Bacillus subtilis*)	85	450
T0281 (hypothetical protein) (1 WHZ)	90	458

**Table 2:** Average results of Met^5^-enkaphalin.

*α*_Annealing_	Chaotic function	Average energy (Kcal/mol)	Processing time (minutes)	Average dRMSD
0.75	(9)	−3.2864	0.2535	0.8877
0.75	(10)	−4.0060	0.2105	0.9467
0.75	(11)	−3.3431	0.2082	0.9017
0.75	(12)	−3.4586	0.2514	0.9380
0.80	(9)	−3.0485	0.2959	0.9130
0.80	(10)	−4.3873	0.2459	0.9197
0.80	(11)	−4.2264	0.2447	0.9123
0.80	(12)	−3.9217	0.2981	0.8927
0.85	(9)	−3.7723	0.4014	0.9160
0.85	(10)	−4.6635	0.3365	0.8857
0.85	(11)	−4.7060	0.3332	0.8757
0.85	(12)	−4.2143	0.3957	0.8910
0.90	(9)	−3.7260	0.5581	0.8963
0.90	(10)	−4.7153	0.4626	0.8827
0.90	(11)	−4.6326	0.4627	0.8987
0.90	(12)	−4.8833	0.5585	0.8953
0.95	(9)	−5.0771	1.3507	0.8957
0.95	(10)	−4.9370	1.1181	0.9137
0.95	(11)	−5.4390	1.1191	0.8913
0.95	(12)	−5.3156	1.3501	0.8963

**Table 3:** Average results of proinsulin.

*α*_Annealing_	Chaotic function	Average energy (Kcal/mol)	Processing time (minutes)	Average dRMSD
0.75	(9)	−93.9999	7.8882	0.9127
0.75	(10)	−97.7679	6.3553	0.8643
0.75	(11)	−101.9142	6.3597	0.8703
0.75	(12)	−95.6412	7.9355	0.8960
0.80	(9)	−96.7255	9.3634	0.8830
0.80	(10)	−103.1905	7.5315	0.8847
0.80	(11)	−95.8967	7.5401	0.9290
0.80	(12)	−95.7312	9.3426	0.8920
0.85	(9)	−102.0535	12.0797	0.8523
0.85	(10)	−102.3225	9.7425	0.8893
0.85	(11)	−101.6467	9.7446	0.8590
0.85	(12)	−107.0401	12.1044	0.8933
0.90	(9)	−110.0378	18.3063	0.8427
0.90	(10)	−108.0935	14.7514	0.8503
0.90	(11)	−115.8930	14.7688	0.8503
0.90	(12)	−110.3555	18.3217	0.8310
0.95	(9)	−120.7662	47.1712	0.8503
0.95	(10)	−121.2029	38.0359	0.8550
0.95	(11)	−126.9481	38.0507	0.8233
0.95	(12)	−122.4787	47.2287	0.8240

**Table 4:** Average results of T0549 instance.

*α*_Annealing_	Chaotic function	Average energy (Kcal/mol)	Processing time (minutes)	Average dRMSD
0.75	(9)	−180.1067	57.3851	0.7787
0.75	(10)	−184.8485	45.5662	0.8820
0.75	(11)	−188.2290	46.2738	0.8567
0.75	(12)	−187.8759	58.0133	0.8077
0.80	(9)	−187.5483	64.8901	0.8887
0.80	(10)	−194.7957	52.4376	0.8333
0.80	(11)	−204.7029	52.9562	0.8333
0.80	(12)	−194.0105	65.0954	0.7963
0.85	(9)	−212.1957	80.4466	0.7827
0.85	(10)	−213.3221	64.6595	0.8300
0.85	(11)	−220.9546	64.9489	0.8423
0.85	(12)	−212.0730	80.7839	0.8763
0.90	(9)	−236.1182	117.9315	0.8190
0.90	(10)	−241.1672	94.6274	0.8143
0.90	(11)	−230.1859	94.9217	0.8357
0.90	(12)	−230.0091	117.9894	0.8093
0.95	(9)	−269.6413	288.8558	0.7200
0.95	(10)	−263.9817	232.2564	0.7643
0.95	(11)	−262.1850	232.2011	0.8203
0.95	(12)	−262.4749	289.0106	0.8123

**Table 5:** Average results of T0335 instance.

*α*_Annealing_	Chaotic function	Average energy (Kcal/mol)	Processing time (minutes)	Average dRMSD
0.75	(9)	−267.9740	103.6328	1.0507
0.75	(10)	−273.8770	82.4738	0.9760
0.75	(11)	−270.0242	82.5450	1.0387
0.75	(12)	−281.0588	102.7148	0.9503
0.80	(9)	−285.2499	114.5852	0.9963
0.80	(10)	−293.6892	89.3586	0.9707
0.80	(11)	−287.6764	89.1567	0.9360
0.80	(12)	−296.9811	113.4518	1.0023
0.85	(9)	−305.8353	135.3040	1.0110
0.85	(10)	−305.2537	107.3173	1.0560
0.85	(11)	−300.5720	108.3275	0.9677
0.85	(12)	−300.6663	134.0739	0.9247
0.90	(9)	−329.4824	194.3791	0.9960
0.90	(10)	−334.7426	155.6107	0.8840
0.90	(11)	−327.1407	155.8174	0.9440
0.90	(12)	−324.0686	194.1348	0.9017
0.95	(9)	−368.4190	473.1948	0.9777
0.95	(10)	−377.6919	379.8146	0.9787
0.95	(11)	−372.4837	380.0762	0.9203
0.95	(12)	−375.3686	473.5348	1.0157

**Table 6:** Average results of T0281 instance.

*α*_Annealing_	Chaotic function	Average energy (Kcal/mol)	Processing time (minutes)	Average dRMSD
0.75	(9)	−206.5214	110.4517	2.9467
0.75	(10)	−215.2062	84.8609	2.9493
0.75	(11)	−205.6181	84.4000	2.7670
0.75	(12)	−211.9487	107.4365	2.9520
0.80	(9)	−224.1211	123.1385	2.9457
0.80	(10)	−233.2700	96.1731	2.9877
0.80	(11)	−223.8302	96.5483	2.9027
0.80	(12)	−222.5050	123.2675	2.8690
0.85	(9)	−251.9814	150.0813	2.9390
0.85	(10)	−245.7741	120.1284	2.8303
0.85	(11)	−251.0341	120.1144	2.8810
0.85	(12)	−259.9042	149.6338	2.8929
0.90	(9)	−273.9763	221.5904	2.8367
0.90	(10)	−260.1847	177.4887	2.8740
0.90	(11)	−281.4230	177.3376	2.9937
0.90	(12)	−290.0598	221.0355	2.8157
0.95	(9)	−314.9119	554.1073	3.000
0.95	(10)	−310.1975	444.6089	2.8633
0.95	(11)	−319.7511	444.5729	2.9873
0.95	(12)	−319.9603	554.1053	2.9197

**Table 7:** Comparison of results with other implementations.

Instance	Approach	Average energy (Kcal/mol)	Processing time (minutes)	Average dRMSD
Met	CMQA	−5.1922	1.2345	0.8993
Met	MQA plus DEP	−0.3775	2.8278	0.8883
Met	CSA	20.0864	0.0593	1.0267
Proinsulin	CMQA	−122.8490	42.6216	0.8382
Proinsulin	MQA plus DEP	−120.6576	24.8549	0.8357
Proinsulin	CSA	480.2667	1.9144	1.3263
T0549	CMQA	−264.5707	260.5810	0.7793
T0549	MQA plus DEP	−259.5423	187.5398	0.7277
T0549	CSA	1795.7408	12.9269	1.4320
T0335	CMQA	−373.4908	426.6551	0.9731
T0335	MQA plus DEP	−298.4703	130.3261	1.0453
T0335	CSA	3745.1859	3.3071	1.3413
T0281	CMQA	−316.2052	499.3486	2.9426
T0281	MQA plus DEP	−310.6578	407.8754	2.7654
T0281	CSA	2998.1609	22.6357	3.1280
